# DJ-1 attenuates the glycation of mitochondrial complex I and complex III in the post-ischemic heart

**DOI:** 10.1038/s41598-021-98722-1

**Published:** 2021-09-30

**Authors:** Yvanna Pantner, Rohini Polavarapu, Lih-Shen Chin, Lian Li, Yuuki Shimizu, John W. Calvert

**Affiliations:** 1 Department of Surgery, Division of Cardiothoracic Surgery, Carlyle Fraser Heart Center, Emory University School of Medicine, 101 Woodruff Circle, Atlanta, GA 30322 USA; 2 Department Pharmacology, Emory University School of Medicine, Atlanta, GA USA; 3 Department of Cardiology, Nagoya University Graduate School of Medicine, Nagoya, 466-8550 Japan

**Keywords:** Stress signalling, Heart failure

## Abstract

DJ-1 is a ubiquitously expressed protein that protects cells from stress through its conversion into an active protease. Recent work found that the active form of DJ-1 was induced in the ischemic heart as an endogenous mechanism to attenuate glycative stress—the non-enzymatic glycosylation of proteins. However, specific proteins protected from glycative stress by DJ-1 are not known. Given that mitochondrial electron transport proteins have a propensity for being targets of glycative stress, we investigated if DJ-1 regulates the glycation of Complex I and Complex III after myocardial ischemia–reperfusion (I/R) injury. Initial studies found that DJ-1 localized to the mitochondria and increased its interaction with Complex I and Complex III 3 days after the onset of myocardial I/R injury. Next, we investigated the role DJ-1 plays in modulating glycative stress in the mitochondria. Analysis revealed that compared to wild-type control mice, mitochondria from DJ-1 deficient (DJ-1 KO) hearts showed increased levels of glycative stress following I/R. Additionally, Complex I and Complex III glycation were found to be at higher levels in DJ-1 KO hearts. This corresponded with reduced complex activities, as well as reduced mitochondrial oxygen consumption ant ATP synthesis in the presence of pyruvate and malate. To further determine if DJ-1 influenced the glycation of the complexes, an adenoviral approach was used to over-express the active form of DJ-1(AAV9-DJ1ΔC). Under I/R conditions, the glycation of Complex I and Complex III were attenuated in hearts treated with AAV9-DJ1ΔC. This was accompanied by improvements in complex activities, oxygen consumption, and ATP production. Together, this data suggests that cardiac DJ-1 maintains Complex I and Complex III efficiency and mitochondrial function during the recovery from I/R injury. In elucidating a specific mechanism for DJ-1’s role in the post-ischemic heart, these data break new ground for potential therapeutic strategies using DJ-1 as a target.

## Introduction

Ischemic heart disease continues to have a high burden of disease globally^1^. Myocardial ischemia–reperfusion injury (I/R) is a major determining factor of the severity of ischemic heart disease^2^. Despite decades of research on therapeutic interventions for myocardial I/R, few have made it to the clinic^3^. The sheer complexity of the cellular processes that occur during the onset and progression of myocardial I/R is a major barrier to successful translational therapies. There is a deficit in the understanding of the biological mechanisms that occur in the hours and days following the onset myocardial I/R injury^4^. Thus, it is critical to investigate the cell signaling systems that are still not fully elucidated.

DJ-1 is a cytoprotective protein that has been shown to play an important role in multiple cellular processes^5,6^. Early examination discovered that mutations and oxidative damage to the protein were associated with early-onset Parkinson’s disease. Thus, DJ-1 is most well-characterized in the brain, where it has been revealed to have anti-oxidant and anti-apoptotic properties^6–8^. DJ-1 shares sequence homology with a family of bacterial proteases and with an *E. coli* chaperone that possesses protease activity^8^. Proteases regulate cellular processes by catalyzing the cleavage of peptide bonds in proteins. Proteases generally reside in cells as latent precursors called zymogens so as to avoid potentially hazardous consequences of unregulated protease activity. In response to stimuli the zymogen is converted into an active protease. Evidence shows that DJ-1 possesses proteolytic activity^9^. More specifically, under oxidative stress conditions, DJ-1 undergoes C-terminal cleavage. This cleavage removes a 15-amino acid peptide, and converts DJ-1 into a protease^8^. The cleaved form of DJ-1 has 26-fold higher proteolytic activity compared to the full length form^8^. Our previous studies have shown that DJ-1 and its cleaved form play a critical role in the heart. Specifically, DJ-1 has been shown to protect the heart in models of acute myocardial infarction and ischemic-induced heart failure^10,11^.

Glycative stress describes the disruption of cell homeostasis by an accumulation of reactive carbonyls^12^. This condition is initiated by non-enzymatic glycosylation known as the Maillard reaction—a complex process that begins when reducing sugars bind to free amino groups in proteins, nucleotides, and lipids^13^. When occurring endogenously, this reaction is termed glycation. Thus, the compounds formed in the advanced stages of this reaction, such as carboxymethyllysine (CML), are called advanced glycation end products (AGEs)^14,15^. AGEs and their precursor dicarbonyls have been shown to be pro-inflammatory and pro-oxidant compounds^16^. When upregulated by AGE, the receptor for AGE (RAGE) intensifies oxidative stress by prompting increased reactive oxygen species (ROS) production^17,18^. Studies have linked the AGE-RAGE pathway to myocardial ischemic injury-associated cardiac dysfunction^17,19,20^. Recent work indicates that through its proteolytic activity, DJ-1 decreases glycative stress^17,19,20^. Although DJ-1 has been shown to reduce glycative stress in the heart^11^, the specific cellular substrates that DJ-1 protects from glycation are still unknown.

Glycative stress has been implicated in various cellular and organelle dysfunction. For instance, the accumulation of dicarbonyls, such as methylglyoxal (MG), in the mitochondria leads to mitochondrial dysfunction^21,22^. Mechanistically, this is thought to involve the glycation and inhibition of respiratory chain complexes^21,23^. In the setting of myocardial I/R injury, there has not been much investigation of the glycation of mitochondrial proteins. As a critical player in the heart’s response to injury, the disruption of mitochondrial function by glycation would likely impact recovery following myocardial I/R. There is evidence that DJ-1 localizes to the mitochondria early after the onset of myocardial I/R injury^10^. However, the physiological consequences for this localization are not understood. Here, we sought to determine if DJ-1 alters protein glycation in mitochondria following the onset myocardial I/R injury.

## Results

### DJ-1 deficiency enhances the accumulation of reactive dicarbonyls in mitochondria

**Figure 1:**
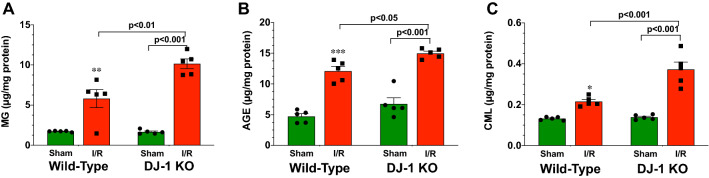
Deficiency of DJ-1 Enhances ischemia–reperfusion induced mitochondrial glycative stress. Levels of (**A**) methylglyoxal (MG), (**B**) advanced glycation end products (AGE), and (**C**) carboxymethyllysine (CML) in mitochondrial fractions. All measurements were performed in samples collected at 3 days of reperfusion from Wild-Type and DJ-1 KO mice. Values are means ± SEM. 5 samples per group. Two-way ANOVA with Tukey’s multiple comparisons test. *p < 0.05, **p < 0.01, and ***p < 0.001 vs. Wild-Type Sham.

Previously, we found that the deficiency of DJ-1 enhances the accumulation of reactive dicarbonyls in hearts following the onset of ischemia–reperfusion injury^11^. However, we did not investigate the accumulation of reactive dicarbonyls in different cellular compartments. Here, we focused on mitochondria given their susceptibility to glycative stress^21,23^. Initial analysis at 3 days of reperfusion revealed significant accumulations of MG, AGE, and CML in mitochondria isolated from the hearts of Wild-Type (WT) mice (Fig. 1A–C). The levels of all three were further increased in mitochondria isolated from the hearts of DJ-1 deficient mice (Fig. 1).

### DJ-1 localizes to the mitochondria and interacts with complex I and complex III after myocardial I/R

**Figure 2:**
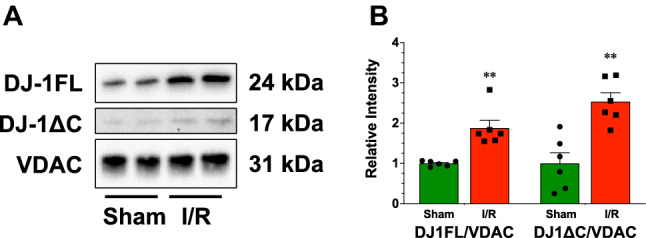
Ischemia–reperfusion injury enhances the localization of DJ-1 to the mitochondria. (**A**) Immunoblots and (**B**) analysis of the full-length (DJ-1FL) and cleaved (DJ-1ΔC) forms of DJ-1 in mitochondrial fractions. All measurements were performed in samples collected at 3 days of reperfusion from Wild-Type mice. Values are means ± SEM. 6 samples per group. Student’s T-test. **p < 0.01 vs. Sham.

**Figure 3:**
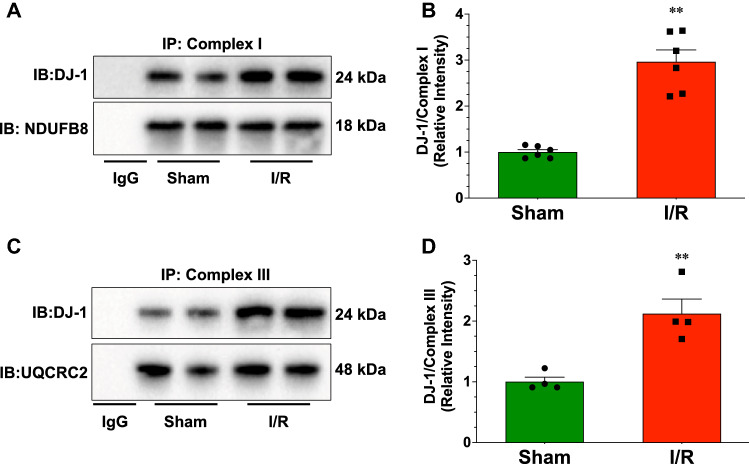
Ischemia–reperfusion injury enhances the interaction of DJ-1 with complex I and complex III of the mitochondrial electron transport chain. (**A**) Immunoblots and (**B**) analysis of the interaction between DJ-1 and complex I of the mitochondria electron transport chain. (**C**) Immunoblots and (**D**) analysis of the interaction between DJ-1 and complex III of the mitochondria electron transport chain. Measurements were performed in samples collected at 3 days of reperfusion from Wild-Type mice. Values are means ± SEM. 4–6 samples per group. Student’s *t* test. **p < 0.01 vs. Sham.

Previous studies indicate the in response to different stimuli, DJ-1 localizes to the inner mitochondrial membrane and mitochondrial matrix^24,25^. Additionally, we previously found that DJ-1 localizes to the mitochondria 4 h after the onset of reperfusion following myocardial ischemia^10^. Here, we found that the expression of the full-length form of DJ-1 (DJ1FL) and the cleaved form (DJ1ΔC) were both elevated in mitochondrial fractions at 3 days of reperfusion (Fig. 2). As noted, the physiological consequence of this localization is not fully understood. There is evidence that DJ-1 interacts with different components of the mitochondria electron transport chain^24^. Therefore, we sought to determine if myocardial I/R induced the interaction of DJ-1 with Complex I and Complex III. For these experiments, we performed separate immunoprecipitation experiments using antibody capture kits to Complex I and Complex III. In both experiments, we found that the interaction of DJ-1 with Complex I and Complex III were increased 3 days post myocardial I/R injury (Fig. 3).

### DJ-1 deficiency enhances glycation of complex I and complex III

**Figure 4:**
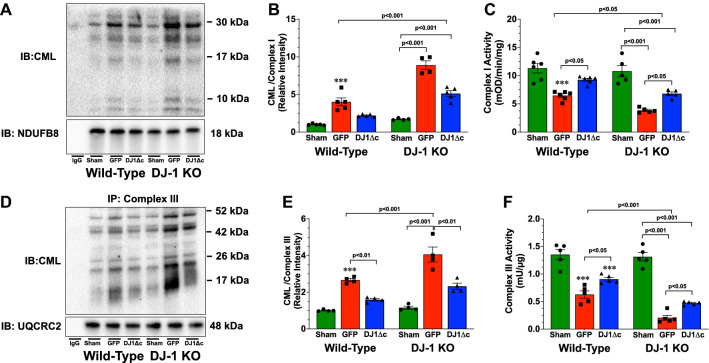
Overexpression of Cleaved form of DJ-1 attenuates ischemia–reperfusion induced glycation of complex I and complex III. (**A**) Immunoblots and (**B**) analysis from immunoprecipitation experiments examining the glycative status of complex I of the mitochondria electron transport chain. (**C**) Complex I activity. **(D**) Immunoblots and (**E**) analysis from immunoprecipitation experiments examining the glycative status of complex III of the mitochondria electron transport chain. (**F**) Complex III activity. Measurements were performed in samples collected at 3 days of reperfusion from wild-type and DJ-1 KO mice administered AAV9-GFP (GFP) or AAV9-DJ1Δc (DJ1Δc). Values are means ± SEM. 4–6 samples per group. Two-way ANOVA with a Tukey test as the posthoc analysis. ***p < 0.001 vs. wild-type Sham. *IP* immunoprecipitation. *IB* immunoblot.

To delve further into the effects of glycative stress on the mitochondria, we again performed separate immunoprecipitation experiments to assess the glycation status of Complex I and Complex III (Fig. 4). Analysis revealed that myocardial I/R injury increased the levels of CML bound to Complex I in the Wild-Type heart (Fig. 4A,B). The levels were further increased in the hearts of DJ-1 KO mice. To further assess the impact of glycation on Complex I, we next evaluated its activity (Fig. 4C). Myocardial I/R injury led to a marked decrease in Complex I activity in the Wild-Type heart. A further decline was observed in the hearts of DJ-1 KO mice. Similar results were observed for Complex III (Fig. 4D–F). Together, this data suggests that the absence of DJ-1 augments the onset of glycative stress in the mitochondria under I/R conditions leading to the glycation and inhibition of Complex I and Complex III.

### Mitochondrial Function is suppressed in DJ-1 knockout hearts after I/R

**Figure 5:**
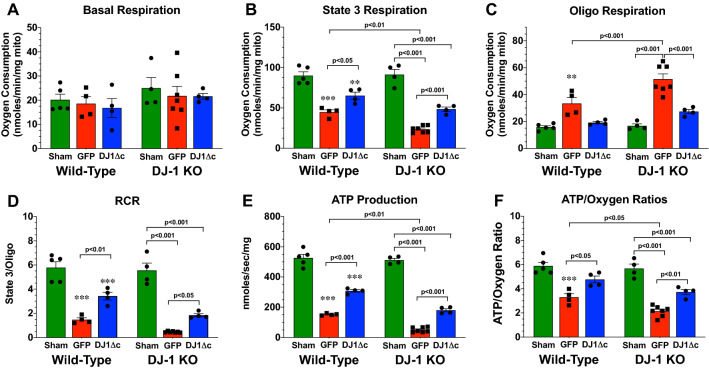
Overexpression of cleaved form of DJ-1 attenuates ischemia–reperfusion induced mitochondrial dysfunction. (**A**) Basal, (**B**) maximum (State 3), and (**C**) oligomycin (Oligo) oxygen consumption rates in the presence of pyruvate and malate. (**D**) Respiratory control ratio (RCR; State 3/oligomycin). (**E**) ATP production rates. (**F**) Efficiency of ATP synthesis [ATP produced per oxygen consumed (ATP/O)]. All measurements were performed in samples collected at 3 days of reperfusion from wild-type and DJ-1 KO mice administered AAV9-GFP (GFP) or AAV9-DJ1Δc (DJ1Δc). Values are means ± SEM. 4–7 samples per group. Two-way ANOVA with a Tukey test as the posthoc analysis. **p < 0.01 and ***p < 0.001 vs. wild-type Sham.

Next, we sought to characterize the mitochondrial functional phenotype of hearts from Wild-Type and DJ-1 KO mice following myocardial I/R injury. Specifically, respiration (oxygen consumption) was assessed using isolated mitochondria. For these experiments, pyruvate and malate were used as substrates. Basal respiration was assessed followed by maximal ADP-stimulated state 3 respiration. Finally, the proportion of uncoupled respiration was assessed by measuring oxygen consumption following the addition of the ATP synthase inhibitor oligomycin, allowing for the calculation of the respiratory control ratio (RCR; state 3 respiration/postoligomycin respiration)^26^. Myocardial I/R did not alter the basal respiration rates of mitochondria isolated from Wild-Type hearts (Fig. 5A). However, a marked decline in maximal ADP-stimulated State 3 respiration rates (Fig. 5B) and a slight increase in oligomycin-stimulated rates (Fig. 5C) were observed. Together this led to a reduction in RCR (Fig. 5D). Basal respiration rates of mitochondria isolated from DJ-1 KO hearts were lower following myocardial I/R compared to Sham levels. Likewise, the deficiency of DJ-1 led to a further I/R-induced reduction in maximal ADP-stimulated State 3 respiration rates, increase in oligomycin-stimulated rates, and reduction in RCR when compared to mitochondria from Wild-Type hearts. Next, maximal rates of ATP synthesis from ADP were assessed during state-3 respiration (Fig. 5E). In agreement with the findings related to respiratory rates, myocardial I/R injury led to a decrease in ATP production rates with lower rates observed in mitochondria isolated from DJ-1 KO hearts. Finally, the ratio of state 3 ATP synthesis rates to state 3 oxygen respiratory rates (ATP/O) confirmed that mitochondria isolated from DJ-1 KO hearts following myocardial I/R injury were less efficient than mitochondrial isolated from Wild-Type hearts (Fig. 5F). Together, this data suggests that the absence of DJ-1 leads to reduced mitochondrial coupling and reduced maximal ATP synthetic capacity in the setting of myocardial I/R injury.

### AAV9-DJ1ΔC attenuates the glycation of complex I and complex III and Improves mitochondrial function

Previously, we found that overexpression of DJ1Δc attenuated myocardial I/R-induced heart failure via the reduction in glycative stress^11^. Here, we asked if the overexpression of DJ1Δc altered the glycation of Complex I and Complex III. For these experiments, WT and DJ1 KO mice treated with AAV9‐DJ1Δc were followed for 2 weeks and then subjected to myocardial I/R. Analysis at 3 days of reperfusion revealed that AAV9-DJ1Δc significantly reduced the glycation of Complex I and Complex III in both strains (Fig. 4). Consistent with a reduction in glycation, the activities of Complex I and Complex III were increased (Fig. 4). With evidence that the overexpression of DJ1Δc reduces glycation of Complex I and Complex III, we next sought to determine the impact of AAV9-DJ1Δc treatment on mitochondrial function. Using a similar approach as outlined above, we found that AAV9‐DJ1Δc significantly improved maximal State-3 respiration, reduced oligomycin-induced respiration, improved rates of ATP synthesis, and improved ATP/O ratios in both strains (Fig. 5). Together this data indicates that the overexpression of DJ1Δc leads to improvements in mitochondrial coupling and maximal ATP synthetic capacity in the setting of myocardial I/R injury.

## Discussion

Mitochondrial dysfunction has been shown to be a major player in the pathogenesis of myocardial I/R injury^27^. The most recognizable role of cardiac mitochondria is the production of energy via ATP synthesis, however, the mitochondria also serve a critical function in mediating cellular homeostasis as they regulate intracellular signaling, calcium storage, fuel utilization, and cell death^28–30^. When normal functions of the mitochondria are disrupted, viability of the entire cell is threatened. Glycative stress contributes to I/R-induced cardiac injury^17,19,20^. However, very little is known about how glycative stress causes cell injury beyond AGE-RAGE signaling-induced pro-apoptotic and pro-inflammatory pathways^18–20,31,32^. In other settings, glycative stress has been shown to have an impact on mitochondrial function^33^. More specifically, treatment of isolated mitochondria or cells with methylglyoxal, glyoxal, of AGEs reduced the activity of respiratory chain complexes, decreased mitochondrial membrane potential, and reduced ATP synthesis^34–38^. Mechanistically, there is some evidence that glycation of Complex III contributes to the impairment of mitochondrial function^23,39^. Here, we found an accumulation of MG, AGE, and CML in mitochondrial fractions 3 days post myocardial I/R injury, suggesting that mitochondria experience glycative stress following the onset of myocardial I/R injury. Moreover, we found that Complex I and Complex III were glycated 3 days post myocardial I/R injury and that this glycation was associated with diminished complex activities and diminished mitochondrial function. Complex I is the largest enzyme complex of the respiratory chain^40^ and some of the most common human oxidative phosphorylation disorders are attributed to Complex I dysfunction^41,42^. Importantly, in response to myocardial I/R injury there is evidence that the activity of Complex I is diminished^43–46^. Complex III is an important component of the electron transport chain, as it independently receives electrons from both Complex I and Complex II^47^. During and after the onset of myocardial ischemia, Complex III activity is depressed, contributing to a reduction in mitochondria respiration^48^. As such, our findings indicate that glycative stress, in part, contributes to the decrease of Complex I and Complex III activities 3 days post myocardial I/R injury.

The evidence for a cardioprotective role for DJ-1 is well established. DJ-1 deficiency has been shown to enhance myocardial infarction and exacerbate left ventricular dysfunction in multiple models including myocardial I/R, permanent myocardial ischemia, and pressure overload-induced heart failure^9,10,49,50^. Further, delayed treatment with the cleaved form of DJ-1 was shown to improve function in mice with myocardial I/R-induced heart failure^11^. However, the exact biological mechanisms behind DJ-1’s role are not well understood. There are many beneficial properties of DJ-1, and it is likely that the advantageous functions of DJ-1 are multi-faceted. DJ-1’s ability to reduce glycative stress^11^ is of particular interest given the evidence for enhanced glycative stress in key injuries to the heart^17,19,20^. The cleavage of DJ-1 into an active protease has been shown to be contribute to its anti-glycative actions^11,14,51^. We have previously shown that the cleaved form of DJ-1 is present in the heart as early as 2 h following the onset of reperfusion after ischemia and persists for at least 7 days^10,11^. In the current study, we found that the cleaved form of DJ-1 localizes to the mitochondria 3 days post I/R injury—a critical time where signaling events are important determinants of cardiac remodeling^11,52^. Here, we have provided a potential mechanism through which DJ-1’s anti-glycative actions protects mitochondrial function in the days following the onset of myocardial I/R injury. Specifically, we expanded on these our findings and suggest that DJ-1 opposes the glycative stress at the mitochondria during the recover from myocardial I/R injury. Our study found that in the absence of DJ-1, mitochondria experienced an enhanced accumulation of reactive dicarbonyls that was associated with depressed mitochondrial function. Mechanistically, we found that DJ-1 interacted with Complex I and Complex III following the onset of I/R injury. This interaction was found to be important in protecting them from glycation, as evidenced by the findings that the glycation of both complexes was exacerbated in hearts of DJ-1 deficient mice. This is further supported by the findings that overexpression of the active form of DJ-1 attenuates the glycation and inactivation of the complexes. Based on this evidence, we hypothesize that during the recovery from myocardial I/R injury DJ-1 maintains the activity of Complex I and Complex III by shielding them from glycation. In turn, this preserves mitochondrial function and reduces cardiac injury^11^.

There is disagreement in the field over the exact mechanism by which DJ-1 protects against glycation. There is evidence to suggest that DJ-1 acts as a glutathione-independent glyoxalase^14^. As a glyoxalase, the mechanism by which DJ-1 acts to diminish glycative stress would include metabolizing the reactive dicarbonyls, thereby halting the formation of AGEs in the mitochondria. Alternatively, a few studies have argued that DJ-1 is a deglycase rather than a glyoxylase^51,53^. As a deglycase, the proposed mechanism by which DJ-1 acts to diminish glycative stress would include the binding to specific proteins and removing the glycation moiety. The findings of the current study tend to support the later mechanism given that DJ-1 directly interacted and altered the glycation of Complex I and Complex III. However, we cannot rule out the possibility that DJ-1 also acts in some capacity as a glyoxalase to reduce the levels of reactive dicarbonyls.

While our current study focused on the ability of DJ‐1 to alter glycation of Complex I and Complex III, there are other mechanisms of action whereby DJ-1 can offer protection during the recover from myocardial I/R injury. First, given the evidence that DJ-1 interacts with Complex V^24^ it is possible that DJ-1 can also shield it from glycation. Second, by reducing glycative stress, DJ-1 could indirectly improve mitochondrial function. Finally, DJ-1 has been reported to influence transcription factors, bind to RNA, alter mitochondria morphology, impact mitophagy and reduce apoptosis^54^. All of these factors could contribute to the protective effects of DJ-1 and indirectly affect mitochondrial function. These alternative hypotheses reveal just how involved and overlapping many of these pathways are. Teasing out the nuances of DJ-1’s protective mechanisms necessitate future study.

Finally, while our current study found that DJ-1 interacted with and altered the glycation of Complex I and Complex III in the setting of myocardial I/R injury, we do know the specific subunits of each complex that are affected. Future studies are therefore warranted to address these interactions.

In summary, this study provides novel evidence that DJ-1 protects Complex I and Complex III from glycation during the recover from myocardial I/R injury. In discerning a specific mechanism for DJ-1’s role in attenuating I/R injury, these data bring us closer to uncovering a therapy that targets the glycative stress pathway in the heart.

## Methods

### Animals

C57BL/6 J mice and DJ-1 deficient (DJ-1 KO) mice^10^ (Male; 8–12 weeks of age) were used in all experiments. Gender influences the development of cardiovascular disease^55^. As such, we only used male mice in our studies. This allowed for the evaluation of DJ-1 in a well-controlled experimental system. All experimental protocols were approved by the Institute for Animal Care and Use Committee at Emory University and conformed to the Guide for the Care and Use of Laboratory Animals, published by the National Institutes of Health (NIH Publication No. 86–23, revised 1996), and with federal and state regulations. Approximately 175 mice were included in the present study after accounting for animal deaths. All mice were randomly assigned to the treatment groups. No animals were excluded from the study. All animal experiments were conductance in accordance with the ARRIVE guidelines.

### Myocardial ischemia–reperfusion injury

Myocardial Ischemia–reperfusion injury was induced by subjecting mice to 60 min of LCA occlusion followed by reperfusion for up to 4 weeks. Surgical ligation of the LCA was performed under anesthesia (ketamine, 100 mg/kg; sodium pentobarbital, 20 mg/kg) as previously described^56^. All animals received prophylactic antibiotic therapy with cefazolin (20 mg/kg) and buprenorphine (0.05 mg/kg) for pain.

### Production of adeno-associated viruses

Plasmid containing a truncated form of human DJ-1 lacking the C-terminal 15 amino acids (DJ1Δc) has been previously described^8^. The DJ1Δc cDNAs was used to generate the recombinant adeno-associated viral expression vector for expression of cleaved human DJ-1 (AAV9-DJ1Δc) under the control of the cytomegalovirus (CMV) promoter. pAAV2/9 containing AAV2 rep and AAV9 capsid genes was kindly provided by the Penn Vector Core (University of Pennsylvania School of Medicine). Recombinant AAV-DJ1Δc viruses were produced by Emory Viral Vector Core, using the triple transfection method with HEK 293 T cells as previously described^57^. The extracted recombinant AAV9 viruses were purified by an iodixanol gradient and was dialyzed using an Amicon 15 100,000MWCO concentration unit. The titer was determined by quantitative polymerase chain reaction. AAV9-GFP also was packaged and used as a control.

### AAV9 infection

Mice were injected with 2 × 10^11^ GC of AAV9-GFP or AAV9-DJ1Δc in 50 µL of PBS 2 weeks before myocardial ischemia via femoral vein injections.

### Western blot analysis

Mitochondrial fractions were obtained using the Mitochondria Isolation Kit for Tissue (89801, ThermoFisher Scientific, Waltham, MA). Protein concentrations were measured with the DC protein assay (Bio-Rad Laboratories, Hercules, CA, USA). Equal amounts of protein were loaded into lanes of Criterion TGX (Tris–Glycine eXtended) Stain-Free PAGE gels (Bio-Rad Laboratories, Hercules, CA, USA. The gels were electrophoresed and activated using a ChemiDoc MP Visualization System (Bio-Rad Laboratories, Hercules, CA, USA). The protein was then transferred to a PVDF membrane. The membranes were then imaged using a ChemiDoc MP Visualization System to obtain an assessment of proper transfer and to obtain total protein loads. The membranes were then blocked and probed with primary antibodies overnight at 4 °C. Immunoblots were next processed with secondary antibodies (Cell Signaling) for 1 h at room temperature. Immunoblots were then probed with a Super Signal West Dura kit (Thermo Fisher Scientific) to visualize signal, followed by visualization using a ChemiDoc MP Visualization System (Bio-Rad Laboratories, Hercules, CA, USA). Data were analyzed using Image Lab (Bio-Rad Laboratories, Hercules, CA, USA)^58^. Uncropped images of immunoblots are depicted in Supplemental Figs. 1–5.

### Immunoprecipitation of complex I

Complex I was immunoprecipitated using the Complex I Immunocapture Kit (ab109711) according to the manufacture’s instructions (Abcam, Cambridge, MA). The samples were then subjected to standard Western blot techniques and the membranes probed with antibodies to CML to assess the glycative status of Complex I and to DJ-1 to assess the interaction of Complex I with DJ-1. An antibody to NDUFB8, a component of Complex I, was used to assess the levels of Complex I.

### Immunoprecipitation of Complex III

Complex III was immunoprecipitated using the Complex I Immunocapture Kit (ab109800) according to the manufacture’s instructions (Abcam, Cambridge, MA). The samples were then subjected to standard Western blot techniques and the membranes probed with antibodies to CML to assess the glycative status of Complex III and to DJ-1 to assess the interaction of Complex III with DJ-1. An antibody to UQCRC2, a component of Complex III, was used to assess the levels of Complex III.

### Glycative stress

Concentrations of methylglyoxal, advanced glycation end-products, and carboxymethyllysine in the heart tissue or cell homogenates was measured using the OxiSelect Methylglyoxal Competitive ELISA kit (STA-811), OxiSelect Advanced Glycation End Product Competitive ELISA kit (STA-817), and OxiSelect N-epsilon-(Carboxymethyl) Lysine Competitive ELISA kit (STA-816), respectively, according to the manufacture’s (Cell Biolabs, Inc, San Diego, CA, USA) instructions.

### Mitochondrial isolation, mitochondrial respiration and ATP synthesis

Cardiac mitochondria were isolated using the Mitochondria Isolation Kit (MITOISO1) according to the manufacture’s instructions (MilliporeSigma, St. Louis, MO). Oxygen consumption of cardiac mitochondria was measured in a sealed chamber magnetically stirred at 37 °C by using calibrated Clark-type oxygen electrode (Hansatech Instruments, Amesbury, MA) in the presence of glutamate and malate as previously described^58^. Maximal (ADP-stimulated) respiration was measured after the addition of a saturating concentration of ADP (1 mmol/L)^59^. Additionally, respiration in the absence of ADP phosphorylation was determined in the presence of 1 mg/ml oligomycin. Respiratory control ratios were determined as the ratio of oligomycin to state 3 respirations. To evaluate ATP synthesis, aliquots were taken from the respiration chamber over a 1-min period after the addition of ADP. ATP was then quantified with a bioluminescence assay using an ATP determination kit (A-22066; Molecular Probes, Eugene, OR). The ATP/O_2_ ratio was calculated with the state 3 respiratory rate for each sample.

#### Complex I and II/III activity

The activity of mitochondria complexes was evaluated in isolated mitochondria using the Complex I Enzyme Activity Microplate Assay Kit (ab109721, Abcam, Cambridge, MA) and the Mitochondrial Complex III Activity Assay Kit (MAK360, MilliporeSigma, St. Louis, MO) according to the manufactures’ instructions.

#### Statistics

All data are expressed as mean ± SEM. The data were first evaluated for normal distribution using the D’Agostina and Pearson omnibus normality test. Subsequent, statistical significance was evaluated as follows: (1) unpaired Student *t* test for comparison between 2 means; (2) a 2-way ANOVA with a Tukey test as the posthoc analysis for comparison among the means from groups of WT and DJ-1 KO mice. A value of *p* < 0.05 denoted statistical significance and *p* values were two-sided. All statistical analysis was performed using Prism 7 (GraphPad Software Inc).

## Supplementary Information

Supplementary Information.

## Data Availability

Data will be available from the authors on reasonable request.
