# Supplementary material for: DJ-1 attenuates the glycation of mitochondrial complex I and complex III in the post-ischemic heart
Source: Sci Rep. 2021 Sep 30;11:19408. doi: 10.1038/s41598-021-98722-1 (PMC8484662; doi:10.1038/s41598-021-98722-1)
Supplement: Supplementary file 1 — Supplementary Information. [file 41598_2021_98722_MOESM1_ESM.pdf]

DJ-1 Attenuates the Glycation of Mitochondrial Complex I and Complex III in the Post-Ischemic Heart

Short Title: DJ-1 opposes glycation of mitochondrial Complex I and III

Yvanna Pantner, B.S.[1], Rohini Polavarapu, B.S.[1], Lih-Shen Chin, Ph.D.[2], Lian Li, Ph.D.[2], Yuuki Shimizu, M.D., Ph.D.[3], John W. Calvert, Ph.D.[1]

[1]Department of Surgery, Division of Cardiothoracic Surgery, Carlyle Fraser Heart Center, Emory University School of Medicine, Atlanta GA USA

[2]Department Pharmacology, Emory University School of Medicine, Atlanta GA USA

[3]Department of Cardiology, Nagoya University Graduate School of Medicine, Nagoya, Japan 466-8550

Correspondence:

John W. Calvert, Ph.D.
Department of Surgery
Division of Cardiothoracic Surgery
Carlyle Fraser Heart Center
Emory University School of Medicine
101 Woodruff Circle
Atlanta, GA 30322
Phone: 404-251-0663
jcalver@emory.edu

Supplemental Figures

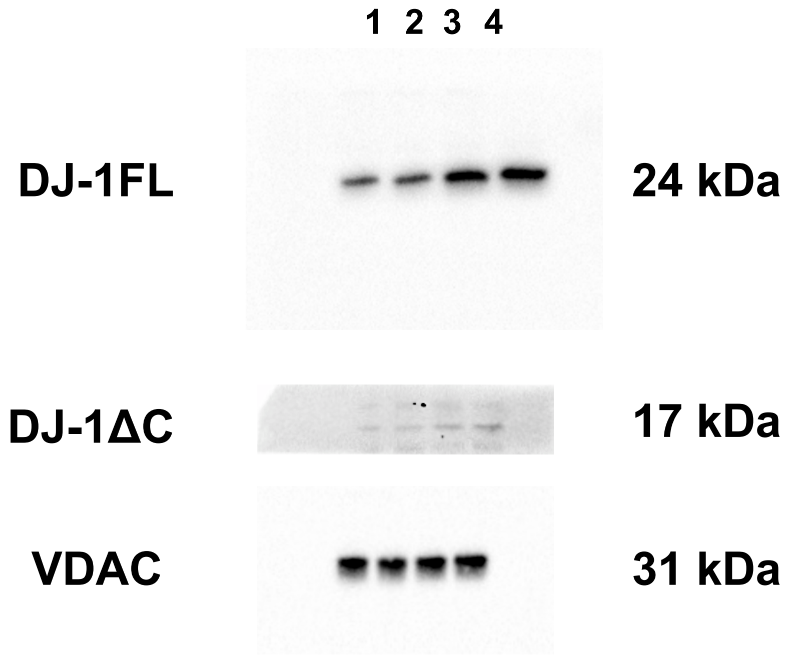

1. Sham
2. Sham
3. IR
4. IR

Supplemental Figure 1. Uncropped images of immunoblots for Figure 2. DJ-1FL, full-length form of DJ-1. DJ-1Δ, cleaved form of DJ-1. IR, ischemia-reperfusion.

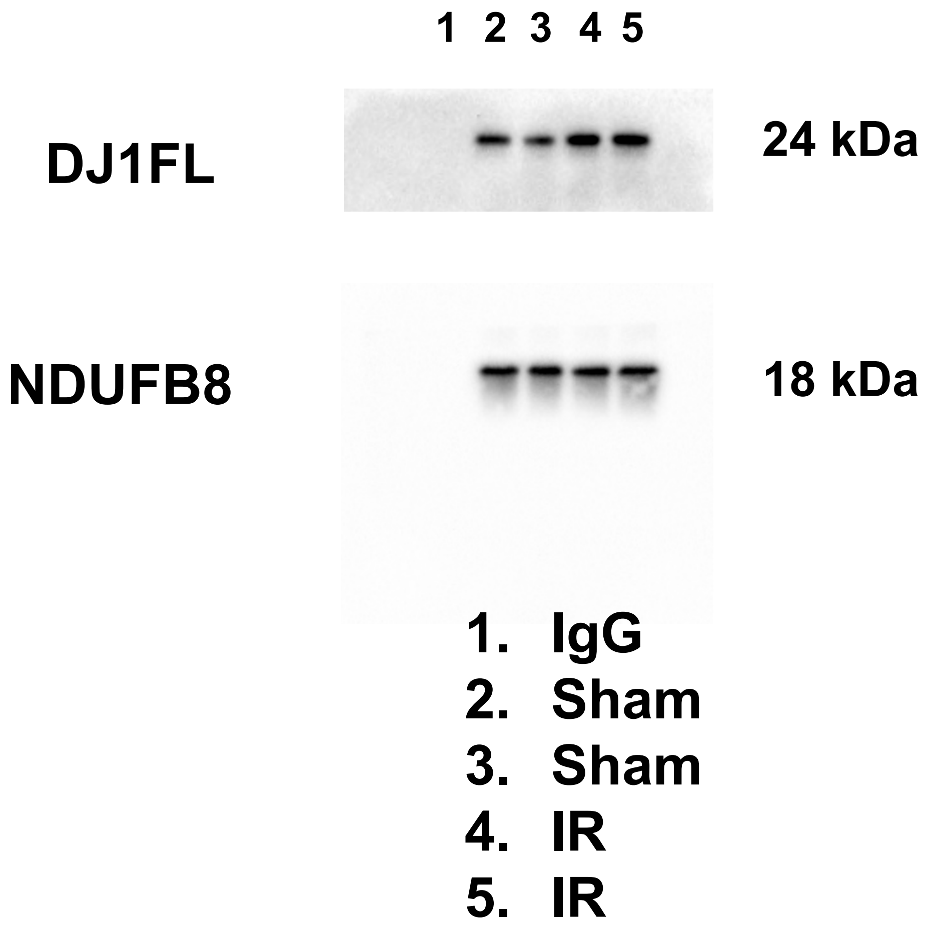

Supplemental Figure 2. Uncropped images of immunoblots for Figure 3A. DJ-1FL, full-length form of DJ-1. IR, ischemia-reperfusion.

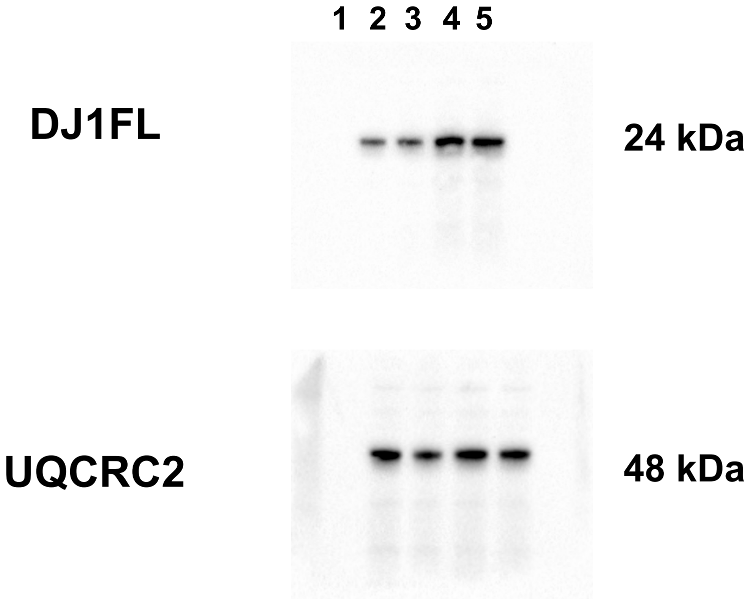

1. IgG
2. Sham
3. Sham
4. IR
5. IR

Supplemental Figure 3. Uncropped images of immunoblots for Figure 3B. DJ-1FL, full-length form of DJ-1. IR, ischemia-reperfusion.

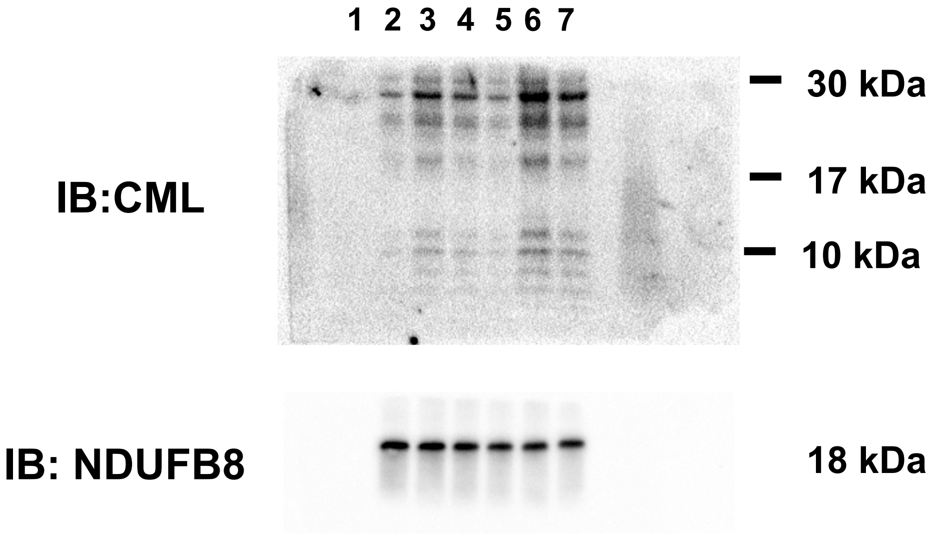

1. **IgG**
2. **WT Sham**
3. **WT IR + GFP**
4. **WT IR + DJ1Δc**
5. **DJ1 KO Sham**
6. **DJ1 KO IR + GFP**
7. **DJ1 KO IR + DJ1Δc**

Supplemental Figure 4. Uncropped images of immunoblots for Figure 4A. DJ-1Δ, cleaved form of DJ-1. IR, ischemia-reperfusion.

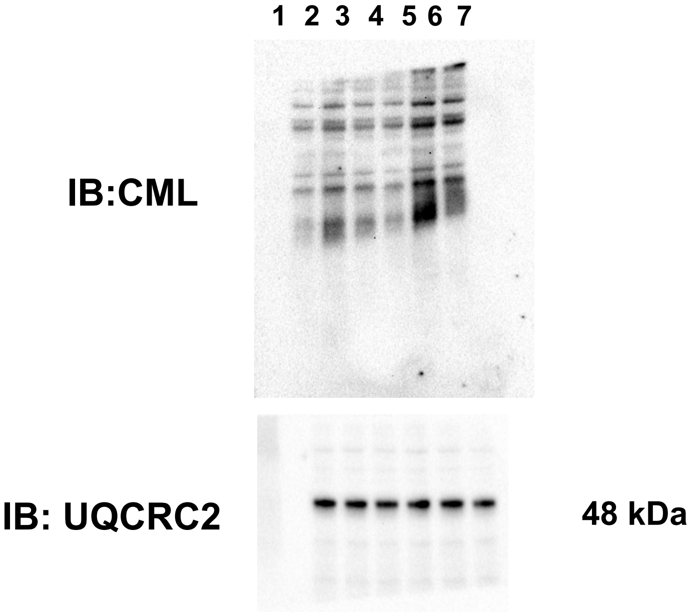

1. IgG
2. WT Sham
3. WT IR + GFP
4. WT IR + DJ1Δc
5. DJ1 KO Sham
6. DJ1 KO IR + GFP
7. DJ1 KO IR + DJ1Δc

Supplemental Figure 5. Uncropped images of immunoblots for Figure 4B. DJ-1Δ, cleaved form of DJ-1. IR, ischemia-reperfusion.
